# Acceptability of youth clubs focusing on comprehensive sexual and reproductive health education in rural Zambian schools: a case of Central Province

**DOI:** 10.1186/s12913-020-4889-0

**Published:** 2020-01-16

**Authors:** Eunice Chirwa-Kambole, Joar Svanemyr, Ingvild Sandøy, Peter Hangoma, Joseph Mumba Zulu

**Affiliations:** 1 School of Public Health, University of Zambia, P.O. Box 50110, Lusaka, Zambia; 2 Chr. Michelsen Institute, Bergen, Norway; 3 Centre for Intervention Science in Maternal and Child Health (CISMAC), Centre for International Health (CIH), University of Bergen, Bergen, Norway

**Keywords:** Acceptability, Youth clubs, Comprehensive sexual and reproductive health education

## Abstract

**Background:**

The youths in Zambia have limited access to information concerning Sexual Reproductive Health (SRH) and this puts them at risk of unwanted pregnancies. Talking about other methods of preventing pregnancy or sexually transmitted infections than abstinence is regarded as culturally unacceptable. The Research Initiative to Support the Empowerment of Girls (RISE) is a cluster randomised controlled trial testing the effectiveness of different support packages on teenage pregnancies, early marriages and school drop-out rates. One of the support packages included youth clubs focusing on Comprehensive Sexual and Reproductive Health Education (CSRHE).

Although similar interventions have been implemented in other settings, their integration process has been complex and comprehensive assessments of factors shaping acceptability of CSRHE are lacking. This article qualitatively aimed at identifying factors that shaped the acceptability of CSRHE youth clubs in rural schools in Central Province.

**Method:**

A qualitative case study was conducted after the youth clubs had been running for a year. Data were gathered through eight focus group discussions with grade eight pupils and eight individual interviews with teachers. Data were analysed using thematic analysis.

**Results:**

The perceived advantage and simplicity of the clubs related to the use of participatory learning methods, films and role plays to communicate sensitive reproductive health information made the learners like the youth clubs. Further, the perceived compatibility of the content of the sessions with the science curriculum increased the learners’ interest in the youth clubs as the meetings also helped them to prepare for the school examinations. However, cultural and religious beliefs among teachers and parents regarding the use of contraceptives complicated the delivery of reproductive health messages and the acceptability of youth clubs’ information among the learners.

**Conclusion:**

The study indicated that CSRHE youth clubs may be acceptable in rural schools if participatory learning methods are used and head-teachers, teachers as well as parents appreciate and support the clubs.

## Background

Every year, an estimated 21 million girls aged 15 to 19 years and 2 million girls aged less than 15 years become pregnant in developing regions [1]. Records show that 46% of girls under the age of 18 years are married in South Asia, 39% in sub-Saharan Africa, 29% in Latin America and the Caribbean, and 18% in the Middle East and North Africa (https://www.girlsnotbrides.org/wp-content/uploads/2014/10/01-Child-marriage-Media-brief-GIRLS-NOT-BRIDES.pdf). In addition, annually 250,000 young people aged 15 to 19 years are newly infected with HIV according to 2015 data [2]. Young people’s limited exposure to Sexual Reproductive Health (SRH) information is among the factors leading to this situation.

Young people in Zambia are faced with a range of problems related to their sexual and reproductive health such as unwanted pregnancies, unsafe abortions, STI/HIV infections and Gender Based Violence (GBV) [3]. However, discussion of subjects such as sexual health and sexuality is still regarded as inappropriate in many areas of the country, especially in rural communities. Therefore, young people in Zambia do not get appropriate guidance on how to avoid pregnancies. This has necessitated the introduction and development of several interventions aimed at improving the SRH among the youth. One such intervention is the provision of Comprehensive Sexual and Reproductive Health education (CSRHE) and life skills education [4].

Zambia is also amongst the 20 hotspots in the world as regards the incidence of child marriage. The overwhelming majority of child marriages, both formal and informal, involve girls under 18 years old, although at times their spouses are also under-age [5]. Of those married, 42% are married before the age of 18 years, and 9% are married before 15 years of age [5]. Girls in the poorest 20% households are five times more likely to be married before the age of 18 years than those in the richest 20% households [5]. Data from the 2013–2014 Demographic and Health Survey and the National Census of Population 2010 (https://zambia.unfpa.org/sites/default/files/pub/pdf/Child%20Marriage%20in%20Zambia.pdf) showed that there had been little change in the national prevalence rate of child marriages since 2002.

Although there has been an increase in the number of countries integrating CSRHE in their education systems, studies on the integration of these programmes into the education systems in low and middle income countries shows that the integration has not been optimal [6, 7]. Meanwhile, there is limited knowledge on the factors that shape the acceptability and adoption of such innovations. This study, therefore, aimed at contributing to this knowledge gap by exploring the acceptability and adoption of CSRHE youth clubs in schools during the implementation of a randomised-controlled trial on interventions that may reduce early childbearing in rural Zambia.

## Methods

### The RISE project

This study was embedded in the Research Initiative to Support the Empowerment of Girls (RISE) study. RISE is a cluster randomised-controlled trial (CRCT) to test the effectiveness of different support packages on teenage pregnancies, early marriages and school drop-out rates. Approximately 4900 girls who were enrolled in grade seven in 2016 in 157 schools of Central and Southern provinces of Zambia were recruited into RISE [8]. The interventions were launched in September 2016 and lasted for 27 months until November 2018. The trial has three arms; one control arm and two intervention arms. In one intervention arm, the participants were offered economic support in the form of monthly cash transfers of ZMW 30, their parents were offered annual grants of ZMW 350, and school fees were paid for those who qualified to grade eight and nine [8]. In the second intervention arm, the same economic support was combined with youth clubs focusing on CSRHE and community dialogue meetings. The youth clubs were established to provide CSRHE among in- and out-of-school adolescent girls and boys. Girls participating in the trial and boys who attended grade seven in 2016 in the randomly selected schools were invited to participate in a youth club every fortnight during the school terms (which are approximately 3 months), and girls and boys could continue in the youth club even if they quit school. The meetings included interactive discussions on education, early marriage, and the risks of early pregnancy, gender roles, and SRH, including myths around modern contraceptives. The premise was that CSRHE information could reduce sexual risk-taking, and life skills and discussions of gender dynamics could make the girls better able to negotiate with boys to delay sex or use protection methods, and thus reduce the risk of early child-bearing. Snacks and a drink were served to those attending the meetings as a way of motivating them to come because the meetings took place in the afternoon when the learners were hungry. Meetings were also held to inform parents about the content of the youth club sessions.

Teachers were linked with a Community Health Assistant (CHA) or a Community Health Worker (CHW) to run the youth club together. Before the intervention was launched, the selected teachers and CHAs/CHWs were given a 5 day training which focused on the SRH curriculum, facilitation techniques and approaches to community mobilisation. Two female peer educators were also recruited per school and trained to assist in mobilising for the youth club meetings and assist with practical things during the meetings. In addition, orientation meetings were held to inform other healthcare workers in the catchment area of the schools about the project and the importance of providing youth-friendly health services.

### Study setting and population

This process evaluation study was conducted in the Central Province of Zambia which is north of the capital city of Lusaka. Central Province is one of the ten Provinces of Zambia. It has 11 districts and a population of 1.3 million [9]. Of this population, 20.9% are youth aged 15 to 24 years [9]. Studies also indicate that Central Province has high levels of teenage pregnancies, early marriages and medium school drop-out rate among the youth [8, 9]. The prevalence of early marriages for the youth in Central Province stands at 46% [9], while the HIV and AIDS prevalence is 12.5%, and the percentage of girls in the age group 13 to 19 years who have begun child-bearing is 29% [10].

### The study site

Central Province has 1120 basic schools [11] of which 1009 are in the rural areas. Basic schools are schools which offer grades one to nine. From the schools in rural areas, RISE offered CSRHE youth clubs in 25 schools.

### Data collection

The interviews were conducted by the first author alone or together with the last author from October to November 2017 in eight different schools. The first author had the experience of working with communities and had conducted action research in a similar community setting previously. This made it easier for the researcher to interact and create rapport with the respondents and facilitated the data collection process.

Before the main interviews were conducted, the interview guide was piloted in one of the districts where the RISE trial was implemented. The feedback from the pilot was discussed with all the authors and the interview guide was refined further. The main tools for data collection are described below in Table 1.

**Table 1:** Data collection elements

Activity	Description
Pilot	First and last author conducted FGD in Chibombo District. FGD guide was tested to check how long FGDs will take. A mixture of English, Bemba and Nyanja were used.
Main study – FGD	A semi-structured FGD guide was used to explore topics such as the overview of CSRHE from pupils, questions on how to prevent early pregnancies, early marriages, school drop-out rates, lessons learnt from the short films, the facilitation of SRH topics by teachers, challenges faced as a result of belonging to the youth club, and if there was any change in behaviour of the pupils. Socio-demographic information, including the date of birth and who the respondents’ guardian were, was also recorded.
Key Informant Interview (KII)	A semi-structured KII guide was used to collect socio-demographic information from key informants such as age, and the following topics were discussed; challenges faced, early marriages, school drop-out rates, early pregnancies, attendance of youth clubs by pupils, inclusion and exclusion of topics, attitude and behaviour of pupils and sustainability of the youth club after RISE.

#### Focus group discussions (FGDs)

A total of eight FGDs were held with pupils, that is, one at each school. The FGDs were conducted in a classroom prepared by the school. The interviews were conducted using a mixture of English and two other local languages namely; Bemba and Nyanja. The interview guide was specifically developed for this study (see Additional file 1) and was translated from English to the two local languages by professional translators. Each FGD consisted of eight participants, that is, four boys and four girls, except for Chibombo, where the pilot study was conducted with 12 participants who were all girls. The FGDs combined both girls and boys since youth clubs were attended by both boys and girls. The purpose of the FGD was to understand the pupils’ perspective of youth clubs.

#### Key informant interviews (KIIs)

KII were conducted with the RISE teacher who facilitated the youth club in each of the eight schools that were visited by the researcher. The interviews were conducted in classrooms and in English since all teachers could speak English. The interviews aimed at understanding the facilitation process of the youth clubs (See Additional file 2).

#### Taking notes

Notes were taken and an audio recording done during the interviews. In the interviews, the notes focused on participants’ behaviour or expressions as well as issues that were interesting and surprising in the interviews and FGDs. The researchers also documented programme-related observations as reported by teachers and pupils such as positive changes and challenges in gender roles.

### Data analysis

All interviews were recorded digitally by the first author and later transcribed verbatim. All the transcripts that were in the local languages were translated into English by the first author. To ensure that content validity was not compromised significantly after the translation, the last author verified the translated transcripts by listening to the audios and comparing them with the transcripts. The authors then familiarised themselves with the data through reading and re-reading the material, noting down initial ideas for analysis. The transcribed data was then carefully read and divided into meaningful analytical units that were relevant to the research aims. By using the method proposed by Zhang and Wildermuth [12] the analytical unit was identified and a code was assigned to signify this particular unit. Each meaningful unit was coded into different sub-categories and then grouped into the major categories that were later framed into themes.

### Ethical issues

Ethical clearance was sought from the University of Zambia Biomedical Research Ethics Committee (UNZABREC) of the University of Zambia Ethics Committee (UNZABREC IRB00001131 of IORG0000774, reference number 061–06-17). Verbal consent was sought from all participants before conducting interviews. Further, a detailed explanation of the research objectives was given to the participants and they were informed that they were free to withdraw at any time. The purpose and nature of the study was explained to the participants. Study participants were assured of anonymity and confidentiality. Participants’ names were not written on the interview schedule and no other person apart from the research team was allowed access to the research data. The audio recordings have been kept by the first author and will be disposed off after a period of 7 years as stated by the University regulations.

## Results

### Socio-demographic characteristics

A total number of 68 youths and eight teachers were interviewed. Pupils’ ages ranged between 13 to 18 years, while teachers were between the ages of 33 to 45 years old. Of the pupils interviewed, 40 were females while 28 were males. Five of the eight teachers interviewed were females while three were males. From these interviews, themes and sub-themes emerged that are listed below in Table 2.

**Table 2:** Selected themes and sub-themes

Code	Major Theme	sub-Theme
A1	Support from parents	Training in line with parental interest
A2	Facilitation	Pupils prefer elderly facilitators to young ones
B1	Change in behaviour	Boys help with house chores Pupils open with parents on SRH

### Advantages of CSRHE youth clubs

In relation to youth clubs, the following were seen by the youth as advantages of the CSRHE provided in the youth clubs; screening of videos showing real life situations, gaining knowledge about SRH, teachers being more supportive than before, and provision of snacks during youth club meetings. Informants explained that they were taught about the realities of life through watching videos. The short films, which depicted real life situations such as teenage pregnancies and complications during delivery, were shown to pupils and this engaged the learners emotionally in the topic. The use of films in reproductive health teaching was a new activity to the learners as none of the previous reproductive health sessions, including science subjects, had adopted films as a teaching mechanism. Teachers also felt that it was a very effective way of teaching. One teacher said pupils did not to forget what they watched in films:

*“There should be more of video-showing because when they know that there will be a video, each and every one will be there. So they are more interested in videos, because they think that is real life. They are able to see what is going on. I think they learnt more from watching videos rather than verbal or group discussions. When you ask a question in relation to the film they will be able to answer everything, so we observed that teaching, seeing and observing is very important. They see it practically other than just talk, but where they watch and discuss, they won’t forget about that”* (KII setting 5).

Informants narrated one of the films which showed the complications that the main character developed due to her young age:

*“Watching videos encourages us not to have early pregnancies because we see that if we fall pregnant whist young, when it’s time to deliver we may have complications, and even after delivery, some people develop the disease called fistula. This comes about when someone is young and they fall pregnant because the bones are not fully developed”* (FGD P5, setting 8).

Informants also said that they had gained knowledge on SRH. At CSRHE youth club meetings, they were taught that certain things which they believed to be true, were myths. One informant said that the teacher had taken time to explain the truth in detail:

*“Some things which people believe to be true are actually not true but myths. Here we are taught the truth. For example, people believe that after having sex for the first time, a woman cannot fall pregnant. We have been taught that actually a woman can fall pregnant even on the first time of having sex. So that is just a myth that a woman cannot fall pregnant”* (FGD P2, setting 3).

One aspect that helped in gaining comprehensive knowledge on sexuality including life skills was the good facilitation by the teachers, CHAs and CHWs that the informants appreciated. Informants felt that the facilitators were knowledgeable about SRH and that all questions and issues related to SRH were answered. The good facilitation skills by facilitators helped informants understand the SRH topics:

*“The way they teach is very good because we understand everything, and they are very helpful. They will never leave any topic hanging but will make sure that we all understand. This also helps us because some topics in science are taught at youth clubs*” (FGD P1, setting 7)*.*

The good facilitation of the programmes led to good mentorship where the pupils felt a sense of ownership and responsibility for the youth clubs and a sense that they have input into what happens there.

*“We feel part of the youth clubs because we feel free to express ourselves without feeling shy. The teachers talk to us with respect”* (FGD P2, setting 1).

Some teachers suggested that the programme should not only target the grade eight but should start from the lower grades:

*“This is a very good programme and we wish that when its scaled up, they will start from the lower classes such as grade three or four so that they start learning about SRH from an early age”* (KII, setting 5).

Another aspect which the youth considered important at youth clubs was the support rendered to them by the teachers, who not only helped materially (mostly applicable to boys, such as buying them books) but also provided counselling. They narrated that teachers supported them so that they could complete school and become independent in future. One pupil said the teacher would feel embarrassed not to help out because the teacher’s aim was to see the pupils complete school and start working:

*“Even the madam (the teacher) encourages us as well that if you have any problems, just come and see me so that I can help you. Some time back, we never used to have this kind of encouragement but now we are being encouraged, even when I approach the madam that I don’t have money for school, she will try to help me because she may feel embarrassed that she has failed to help someone who really wants to learn”* (FGD P8, setting 5).

Informants felt that incentives, such as the snacks that were provided during CSRHE youth clubs’ meetings, encouraged them to continue attending the youth clubs’ meetings:

*“The girls always attend school and they look forward to the youth clubs because of the drinks and biscuits since they know that they will have a drink and a biscuit”* (FGD P2, setting 2)*.*

### Compatibility of CSHRE youth clubs to cultural and social norms

All the youths interviewed preferred elderly facilitators to teach them during youth clubs’ meetings as opposed to young facilitators because according to them, elderly facilitators knew a lot of things and would teach from experience as they had passed through a lot in life. Informants said they preferred facilitators who were above 30 years old. Informants said young facilitators were in the process of learning and would, therefore, not be able to answer some questions due to lack of experience:

*“I would like an elderly facilitator as compared to a young one because the young one may not know most of the things, but the elderly know most of the things of life. The youthful one may feel shy to explain to me in detail like issues to do with sex”* (FGD P4, setting 1).

An aspect that informants were happy with was the support rendered to them by their parents in relation to youth clubs. The informants narrated how their parents were interested in knowing what they learnt from the youth clubs and how they encouraged them not to miss any sessions:

*“Clubs really help us because just when I reach home, my parents usually ask me what I learnt on that particular day, they even remind me when it’s the day for clubs so that I don’t forget to attend”* (FGD P6, setting 4).

According to the teachers, the CSRHE youth clubs would not have succeeded without the support from management. They felt the school management was involved right from the beginning of the programme, where they helped in the sensitisation of parents who did not accept the programme when it began:

*“When the programme started, there was resistance by some parents to enrol their children for the programme, but then the head-teacher would call them for sensitisation meetings”* (KII setting 5)*.*

Teachers said there was a great reduction in school drop-outs rates which was attributed to the CSRHE lessons learnt from the clubs, the monthly allowance which was given to the girls and the school fees paid for them. Many schools that were visited did not report any school drop-outs rates among the RISE participants 1 year after the interventions started, except for one school which recorded an increase in school drop-outs rates. This could have been attributed to the commercial farming block in the area where the youth go to work for economic purposes:

*“Concerning school drop-out, I would say the numbers have reduced as compared to the time before the youth clubs came; before youth clubs started, we had 5 pupils who dropped out but from the time we started last year, no one has dropped out, except that we have recorded one pregnancy this term, though she is still in school”* (KII setting 8).

The youths said they had changed their behaviour after getting lessons from the youth clubs. Some said they had stopped engaging in sexual relationships with the opposite sex as they now knew the dangers of doing that:

*“I used to have relationships with boys but I have since changed, as I have learnt from the youth clubs on the dangers of having sex before marriage”* (FGD P5, setting 5).

Informants said the topics taught during CSRHE youth club meetings were science-related and this made them understand science in class as it was more of revision considering that science was an examinable subject:

*“I learn science from the youth club hence whatever I was supposed to go and study at home, I can learn from the youth meetings. For example, in reproduction, what I am taught here at the youth clubs is what I will find in books when it’s time to study” (*FGD P6, setting 1).

Teachers said that the introduction of the CSRHE youth clubs in schools had a positive impact on the pupils. One teacher said that the class that was once considered consisting of noise makers was now one of the most well behaved in the school and that teachers were wondering what could have happened to the class. They reported that parents were happy as they could see their children take responsibility for a lot of things. One teacher reported that parents were happy with the change they were seeing in their children because even boys now performed work purported to be for girls:

*“One parent said she is happy with what we teach the pupils because some time back before the youth clubs started, boys would refuse to do some house chores saying that the kind of work was for girls, but now they are able to do the work because of what they are learning from the youth clubs”* (KII setting 5).

Informants said they were free to share topics learnt from the clubs with their guardians and other people. Some informants said their parents were interested in knowing what they learnt from the youth clubs. One informant narrated that she was free to tell her parents about what she learned as they were the ones that raised her and hence knew her better. However, the pupils usually preferred to discuss SRH topics with parents/guardians of the same sex:

“ *I can’t feel shy to tell my parents what we learn from RISE because they are the ones that brought me up. I will not choose what to tell my mother and what not to tell her. I can feel shy to tell others in detail but not my parents because they know me very well. Therefore, I should just be comfortable to discuss what we learn from RISE with my parents, though I would be more comfortable with mum than dad because I am female”* (FGD P8, setting 8)*.*

### Challenges

In as much as teachers and pupils were happy with the programme, they also encountered some challenges. One teacher said topics like use of contraceptives did not go well with parents. Some teachers complained that some topics were too sensitive to teach pupils such as the withdrawal method (when having sex) hence some opted not to teach the topic while others just taught it because it was part of the syllabus:

*“The topics they discouraged were where you teach the girls to use contraceptives. Even the parents are not happy with it. It’s there in the module but I haven’t taught them”* (KII setting 5).

According to the youth, they were taught that abstinence was one of the most effective ways which helped prevent early pregnancies and marriages. Informants said engaging in sexual activities would lead to one falling pregnant and they might fail to take care of the child as they were too young. One informant said that girls should not rely on family planning as a way to avoid falling pregnant but instead abstain because in trying to use family planning, one could be given expired drugs and may end up falling pregnant:

*“Girls are not supposed to go for family planning; this is for those that are married and not us who are still in school. Girls should avoid being too familiar with men, that is why men take advantage and ask them out for sex. Then when a girl starts family planning, they may even get drugs that are expired and the day they will decide to have sex with a man, they will fall pregnant. Hence, the best is for girls to keep away from sex”* (FGD P3, setting 7).

However, some girls were in support of using contraceptives as a way of preventing pregnancies and STIs. They felt that those who failed to control their sexual desires should go for family planning. Below is a quote from one of the respondents:

*“Even the teacher mentioned that if you can’t control your sexual feelings, then you can go to the clinic for family planning”* (FGD P8, setting 1).

Another challenge faced by the teachers was in relation to knocking off time as they felt the CSRHE youth club was really demanding as compared to other clubs. Some teachers said they had to come to school as early as six in the morning and knock off late as late as four in the afternoon as they had to take up the CSRHE youth club after classes. One teacher said she lived far from the school and this meant getting home very late:

*“So for now the challenge that I have is time. I am coming from town where my home is, then I report for work at 06:00 hours, then when I finish with class work, I have to wait for the RISE again, then I knock off late about 16:00 hours”* (KII setting 1).

Some teachers said they had work overload as they had many things to do such as preparing for other lessons, and then they needed to find time to go through the RISE manuals as well:

*“I also have class or classes more especially beginning of this term. I was having two classes because I had my class and the other class where I was teaching geography so I had some work overload” (*KII setting1).

Teachers said that the same pupils who attended CSRHE youth club meetings were the same pupils that made up other youth clubs in the school, hence there was a conflict. Some teachers said that they were having challenges because the pupils were expected to attend classes given by another teacher thus postponing the youth meeting to another day:

“*You find that at times we may not have the meetings especially the last two terms that passed because there is that antagonism where some teachers would want to teach the same pupils and I also want the same pupils for CSHRE clubs. Then there may also be other challenges of other clubs where some pupils may be in other clubs, for them to come to RISE, others may not even come because they are needed to some other programmes within the school”* (KII setting 2).

When asked on whether they encountered difficult topics and how they handled such situations, some teachers said they encountered some few difficult topics which needed medical personnel, hence they worked hand in hand with the CHWs. However, sometimes the CHWs who were supposed to help with the health component did not always report for the meetings because of challenges related to distance. In one school, the teacher pointed out that in such situations, she had to take up that subject though she was quick to point out that in such cases the peer educators would help her. The teachers pointed out that the use of manuals made their work easier as they would read through the manuals in cases where the CHWs did not show up:

*“As you can see, the other topics are supposed to be taught by CHWs who are more knowledgeable in those topics. However, the manuals have simplified our work as we read through if the CHW is not around and this has helped us to understand the topics, including those which seem like they are difficulty”* (KII setting 7).

## Discussion

This qualitative study aimed at identifying factors that shaped the acceptability of CSRHE youth clubs in rural schools. The study showed that different issues contributed to the acceptability. The perceived advantage of learning more about topics that could be tested in the science examinations, coupled with good facilitation skills by the teachers, facilitated the acceptability of the CSRHE youth clubs among the learners. The fact that teachers were being more supportive also made pupils accept the youth clubs.

Furthermore, it is also clear that the youth got empowered especially through watching films, and interactive teachings such as group discussions and role plays to helped them understand the dangers of risky sexual behaviour such as having sex at an early age, and the responsibilities of caring for and regularly breastfeeding a baby. Such interactive teaching seems to be more effective as compared to the traditional way since students become engaged in learning and retain more information. This is similar to Senthamar’s [13] write-up from India who stated that the interactive teaching method motivates learning. The Ministry of Education (MoE) should therefore invest more on teaching CSRHE through the use of other teaching methods such as short films in order to effectively reach out to the youth with the important messages.

Having elderly facilitators (teachers and community health workers) who are seen to be knowledgeable on issues of SRH, and topics being in line with parental interest, also positively influenced acceptability of the CSRHE. Teachers play a critical role in the delivery of CSRHE in schools and have an important responsibility to ensure that the youth acquire essential knowledge, skills and attitudes. However, teaching of sexuality education was selective, with some topics being excluded as teachers did not find it compatible to their cultural and religious norms. This may be linked to the teachers’ desire to promote certain moral positions and values. The training in CSRHE of teachers should enable them to appreciate the distinction between their own beliefs and values and what they have learned as separate from the content they are expected to teach during sexuality education. A study conducted by Masinga [14] in South Africa states that if this is not done, it can have a particularly negative effect on sexuality education.

The Zambian Ministry of Education’s (MoE) policy on contraceptives states that there should be no distribution of any type of contraception in schools as it is believed that this would erode the morals of pupils. Teachers and some pupils interviewed also promoted abstinence as the best practice to prevent HIV and AIDS, and school drop-out rates due to early pregnancies and early marriages. However, some female pupils felt that there was need to get contraceptives if they failed to control their sexual desires. In order to have a more effective policy regarding contraceptives, relevant authorities in schools and MoE need to adjust to these developments of pupils’ sexual needs. This requires higher authorities to develop more evidence-based curriculums and guidelines on SRH and rights in general and particularly contraceptives. Teachers should also be trained to improve their skills in discussing SRH-related issues with young people. Jolien et al. [15], state in their study that was carried out in Ghana that there is need for teachers, religious leaders and key figures to have a different approach on SRH issues among the youth in order to have a positive effect on the adolescents’ health.

While Zambia has made progress in increasing CSRHE information in schools, more information is needed on current government programmes and policies to ensure that they are targeting the youth at the right age and providing adequate SRH support. The findings of this study indicate that CSHRE should start as early as possible so that the youth are empowered with life skills to make decisions about their life at an early age. This is in line with a study carried out in Zambia by Menon et al. [16], who also noted that it was important that teaching of life skills start as early as possible so that as children are growing up, they would be able to resist negative pressure and avoid involving themselves in risky behaviour.

The findings indicated that the acceptability of the CSRHE youth clubs in schools where the RISE trial was conducted in Central Province depended partly on whether stakeholders such as pupils and teachers had a sense of ownership. Open communication and sense of respect have been found to contribute to sense of ownership [17]. The use of CHWs and peer educators in youth clubs as well as the involvement of other stakeholders such as health personnel and parents, may affect notions of programme ownership as pupils see different people talk to them on different SRH issues.

Trustworthiness of the study was strengthened through the systematic and analysis of the data and inductively coding and categorisation and through the use of different methods to collect data, that is, focus group discussions, key informant interviews and taking of notes [18]. The researchers also aimed at enhancing credibility and dependability of findings by separately sharing the codes and categories with the co-authors with backgrounds in anthropology and public health. Individual insights of the data were reviewed and discussed to develop the themes. Transferability was further promoted by providing a rich description of the phenomena, informants, the procedures of data collection and analysis, and by providing quotations in the text representing a variety of informants [19].

A limitation of this study was that we conducted only one FGD and one KII and spent 1 day of observations per school. Not including the general community members, CHWs, peer educators, health workers, and policy makers from the educational sector, and limiting the study to grade eight pupils, implied that some important perspectives on the acceptability of CSRHE youth clubs may have been missed. Another limitation was that the study was conducted in Central Province of Zambia using a qualitative approach; hence the findings may not be generalisable to other settings.

## Conclusion

This study has sought to provide an assessment of the acceptability of youth clubs focusing on comprehensive sexual and reproductive health education in the Central Province of Zambia. Our results suggest that pupils and teachers accepted the CSRHE. The advantage and simplicity of the clubs with regard to communicating sensitive reproductive health information through the use of films and role plays compared to other similar programmes influenced acceptability. Most topics taught during the youth clubs were science-related, which is an examinable subject in schools. This situation may benefit the pupils hence accepting the youth clubs as they had a relative advantage in science. However, topics such as contraceptive and condom use affected programme acceptability. For example, teachers deliberately excluded topics on withdrawal method as means to prevent pregnancies. Some CHWs who were supposed to help teachers with certain lessons sometimes did not show up, leaving the teachers with a lot of workload. All in all, this study demonstrates that CSRHE can be provided in an acceptable manner by teachers as long as they are properly trained and are giving interactive teaching materials.

## Supplementary information

**Additional file 1.** FGD interview guide for youth club participants.

**Additional file 2.** Interview guide for teachers.

## Data Availability

The interviews analysed during the current study are available from the corresponding author on reasonable request.

## Abbreviations

CSRHE: Comprehensive sexual and reproductive health education
FGD: Focus group discussion
