# Supplementary material for: Acceptability of youth clubs focusing on comprehensive sexual and reproductive health education in rural Zambian schools: a case of Central Province
Source: BMC Health Serv Res. 2020 Jan 16;20:42. doi: 10.1186/s12913-020-4889-0 (PMC6966797; doi:10.1186/s12913-020-4889-0)
Supplement: Supplementary file 1 — Additional file 1. FGD interview guide for youth club participants. [file 12913_2020_4889_MOESM1_ESM.docx]

**Additional File 1: FGD interview guide for youth club participants**

1. Age
2. Grade
3. Who do you stay with
4. How interesting have the youth clubs been?
5. In what ways have the youth clubs affected early pregnancies in your schools?

(Probe: why do you think so? What is the reason for that? )

1. In what ways have the youth clubs affected early marriages in your schools?

(Probe: why do you think so? What is the reason for that? )

1. What do you think has been most helpful to prevent early marriages among girls at your school? (Probe: why do you think so? What is the reason for that?)
2. In what ways have the youth clubs have changed the way you think about school and education? (Probe: why do you think so? What is the reason for that? )
3. In what ways have the youth clubs have affected the school dropout.
4. What do you think has been most helpful to prevent school dropout among girls at your school? (Probe: why do you think so? What is the reason for that?)
5. Concerning the films that you have watched during your club meetings, what were the key messages you took with you from the films? Do you think they have they been encouraging girls to stay longer in school? (Probe: why do you think so? What is the reason for that? How? In what ways)

**SRH IN YOUTH CLUBS**

1. Youth clubs also looked at topics on SRH: How would you say the topics were facilitated? (Where you comfortable with the facilitator? Further probe: a male or female, age? Give reasons for your answer)
2. How were the sessions organised? How long is each session? Would you prefer that the time is increased or reduced? Give reasons for your answer?
3. Concerning the people leading in club meetings what do you think of the way they deliver the messages (Probe: Why do you say so, if they need to improve, can you suggest ways of how they need to improve? ). How knowledgeable has the person who has been providing you with SRH services been in relation to the topics?
4. Did attending the youth clubs have any conflict in time to study or do other house chores, if so how do you manage such challenges?
5. From what you have learned in the youth clubs, how comfortable are you to discuss the SRH issues with your parents/ guardians? (Probe: among the peers, have your parents noticed any change in your behaviour, has this changed the way you interact with your parents).
