# Supplementary material for: Acceptability of youth clubs focusing on comprehensive sexual and reproductive health education in rural Zambian schools: a case of Central Province
Source: BMC Health Serv Res. 2020 Jan 16;20:42. doi: 10.1186/s12913-020-4889-0 (PMC6966797; doi:10.1186/s12913-020-4889-0)
Supplement: Supplementary file 2 — Additional file 2. Interview guide for teachers. [file 12913_2020_4889_MOESM2_ESM.docx]

**Additional file 2: Interview guide for teachers**

1. Name
2. Age
3. Sex
4. Can you tell us how the youth clubs started in schools (What role did you play)?
5. What challenges do you face when preparing or conducting your class sessions?
6. What do you think has been helpful to prevent early pregnancies among girls at your school? (Why do you say so)
7. In what ways have the youth clubs have affected the school dropout.
8. What do you think has been most helpful to prevent school dropout among girls at your school? (Probe: why do you think so? What is the reason for that?)
9. How many sessions of the youth clubs do you have in a term, how is the attendance from the girls in all the sessions, do you have a register (Check the register of attendance).
10. How has the youth club introduction in schools influenced young people’s attitudes and their thinking? (Probe: their behaviour, and their relationships to friends, boys/girls, family and others in the community?).
11. What are your thoughts about the lessons/ activities in the youth clubs? (What do you feel should be include or excluded, give reasons?).
12. Is the program sustainable even after the funders pull out? Give reasons for your answer
